# Recent advances in multimodal foundation model-enabled peptide screening and optimization for smart biomaterials and functional tissue engineering

**DOI:** 10.3389/fbioe.2026.1884032

**Published:** 2026-07-29

**Authors:** Chen Ding, Yuxi Luo, Ximiao Yu, Yingying Lei, Wenping Zhang, Xun Wang, Jiadi Gan

**Affiliations:** 1 Department of Pulmonary and Critical Care Medicine, The Affiliated Wuxi No. 2 People’s Hospital of Nanjing Medical University, Wuxi, China; 2 Department of Medical Oncology, National Cancer Center/National Clinical Research Center for Cancer/Cancer Hospital, Chinese Academy of Medical Sciences and Peking Union Medical College, Beijing, China; 3 Department of Respiratory and Critical Care Medicine, Affiliated Hospital of Jiangnan University, Wuxi, China; 4 Wuxi School of Medicine, Jiangnan University, Wuxi, China; 5 Department of Neurology, The Second Clinical Medical College, Ningxia Medical University, Yinchuan, China; 6 Department of Orthopaedics, Shengli Oilfield Central Hospital, Dongying, China; 7 Department of Pulmonary and Critical Care Medicine, Frontiers Science Center for Disease-Related Molecular Network, State Key Laboratory of Respiratory Health and Multimorbidity, West China Hospital, West China School of Medicine, Sichuan University, Chengdu, China

**Keywords:** 3D bioprinting, hydrogel bioinks, peptide design, protein language models, regenerative medicine, smart biomaterials

## Abstract

Peptides are important bioactive molecules for smart biomaterials and functional tissue engineering because they can provide targeting, antimicrobial, adhesive, immunomodulatory, and differentiation-regulating functions. However, conventional peptide discovery remains limited by high experimental cost, incomplete exploration of sequence space, and the difficulty of balancing bioactivity with stability, safety, manufacturability, and material compatibility. These limitations become more pronounced when peptides must retain function after chemical modification, immobilization, hydrogel incorporation, crosslinking, printing, or construct maturation. Recent advances in protein language models and multimodal foundation models provide new opportunities for peptide screening, generation, property prediction, and material-aware prioritization. This review summarizes foundation model-assisted strategies for peptide representation learning, candidate retrieval, *de novo* generation, interaction and property prediction, multi-objective optimization, benchmarking, developability assessment, and staged material-level validation. We also discuss representative applications in antimicrobial biomaterials, targeting systems, immunomodulatory materials, peptide-functionalized hydrogels, hydrogel bioinks, and three-dimensional bioprinted constructs. Overall, foundation model-assisted peptide design should be viewed as a candidate-prioritization framework rather than a substitute for experimental validation. Its future value will depend on standardized material-context data, transparent benchmarking, and validation across solution, material, biofabrication, and construct-level settings.

## 1 Introduction

Peptides are short amino acid sequences with broad relevance to biomedical research, biomaterials engineering, and regenerative medicine (Wang et al., 2022; Binaymotlagh et al., 2023). Their target specificity, chemical modifiability, design flexibility, and generally favorable safety profiles position them between small molecules and larger biologics (Venneti and Stockdill, 2023). Beyond therapeutic use, peptides can function as receptor ligands, antimicrobial agents, targeting sequences, adhesion motifs, immune-regulatory cues, or material-functionalization modules (Kumar et al., 2023; Morwood et al., 2023; Xiao et al., 2025). These properties have expanded their use in hydrogels, scaffolds, bioinks, additive manufacturing, and functional tissue-engineering constructs (Kwan et al., 2025; Ma et al., 2025).

Biological activity alone, however, does not determine whether a peptide is suitable for biomaterial applications. Candidate peptides must also remain stable, soluble, manufacturable, selective, and safe (Fetse et al., 2023; Elango and Zamora-Ledezma, 2025). Their apparent function may change after immobilization, hydrogel incorporation, surface conjugation, crosslinking, or printing, and may also depend on matrix stiffness, degradation, ligand density, and spatial presentation (Li L. et al., 2025). Peptide design in this setting is therefore not a single-objective search for maximal activity. It requires a context-dependent balance between molecular function, processing tolerance, material integration, and performance in the final construct (Kaspar and Reichert, 2013; Zhang Z. et al., 2025).

Conventional strategies include high-throughput screening, display technologies, combinatorial libraries, rational design, docking, and molecular dynamics simulations (Fan et al., 2024). These approaches remain essential for motif discovery, mechanistic validation, and final candidate confirmation, but they are costly, time-consuming, and limited in their ability to explore large sequence spaces. Rational and structure-based methods depend on prior knowledge and reliable structures (Yin et al., 2024). These limitations become more consequential when a selected sequence must also tolerate formulation, hydrogel incorporation, surface conjugation, printing, and crosslinking (Lee et al., 2024; Resende et al., 2025). A peptide that performs well in solution may therefore be unsuitable once transferred into a material system.

Artificial intelligence (AI), particularly protein language models (PLMs), sequence-level foundation models (FMs), and emerging multimodal FMs, offers new approaches to peptide screening, generation, and material-aware optimization (Goles et al., 2024; Liu et al., 2025c; Leclercq and Droit, 2026). In this review, the phrase “multimodal foundation model-enabled” in the title refers to a developing direction and organizing framework for integrating sequence, structure, function, material parameters, and experimental readouts. Most current applications, however, still rely mainly on PLM-based models, sequence-level foundation model-assisted workflows, or PLMs combined with task-specific downstream predictors rather than fully integrated multimodal FMs (Elnaggar et al., 2022; Lin et al., 2023). Their practical value lies in transferring information learned from large protein or peptide datasets to tasks with limited labeled examples, while narrowing the experimental search space.

For smart biomaterials and tissue engineering, sequence-derived representations must ultimately be connected with material-context information. Relevant variables include chemical modification, conjugation chemistry, hydrogel formulation, scaffold properties, crosslinking conditions, printing parameters, cellular responses, and construct-level outcomes (Geevarghese et al., 2025; Li L. et al., 2025). Without these variables, a model may predict molecular activity but fail to identify peptides that remain functional after incorporation into a biomaterial system. Material-aware peptide design therefore requires not only stronger models, but also staged validation, transparent benchmarking, and standardized reporting of peptide, material, processing, and biological metadata (Collins et al., 2024; Geevarghese et al., 2025).

This review summarizes recent advances in foundation model-assisted peptide screening, generation, and material-aware optimization for smart biomaterials and functional tissue engineering. We first discuss conventional peptide discovery and the shift toward material-aware workflows. We then examine PLM-based sequence representation learning, multimodal modeling, candidate retrieval, *de novo* generation, discriminative prediction, preference optimization, hybrid pipelines, and design–test–learn strategies. The later sections focus on evaluation metrics, validation hierarchy, developability, material compatibility, biofabrication tolerance, representative application domains, reporting standards, and future directions.

## 2 Conventional peptide discovery and the shift toward material-aware foundation model-assisted workflows

Functional peptide discovery has traditionally relied on experimental screening, display technologies, empirical optimization, and structure-guided design (Jaroszewicz et al., 2022; Hutchings and Sato, 2024; Vincenzi et al., 2024). These approaches remain essential for identifying active motifs, validating mechanisms, and confirming final candidates. However, they are labor-intensive, explore only a limited portion of peptide sequence space, and often struggle to optimize activity, selectivity, stability, safety, and manufacturability at the same time (Liu et al., 2025b). These limitations become more apparent when peptide candidates must be selected for complex biomaterial applications rather than for soluble activity alone.

**FIGURE 1:**
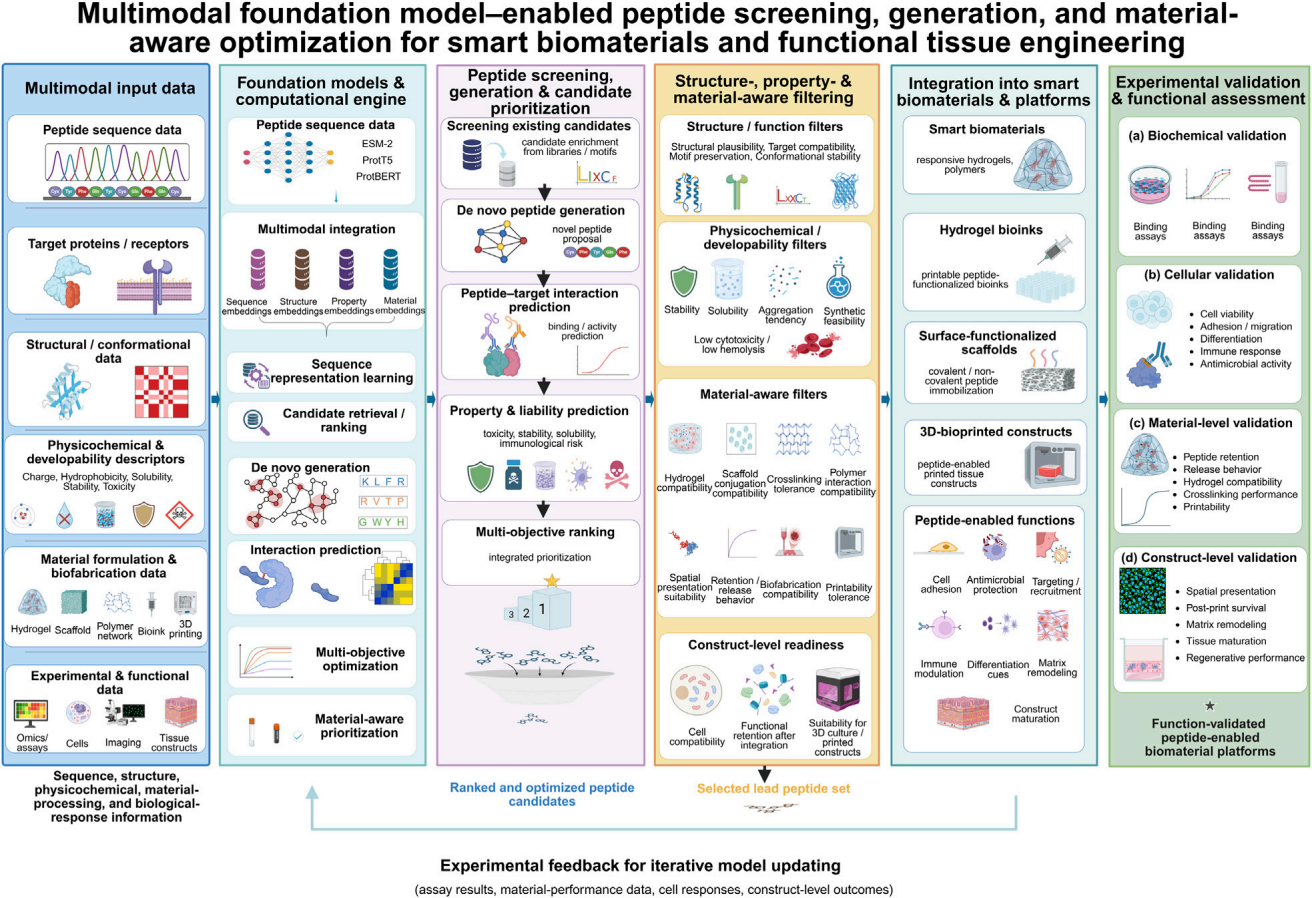
Multimodal foundation model-enabled peptide screening, generation, and material-aware optimization for smart biomaterials and functional tissue engineering. The workflow links multimodal input data, including peptide sequences, targets, structures, physicochemical descriptors, material formulations, biofabrication variables, and experimental readouts, with foundation model-based representation learning and candidate prioritization. Candidate peptides are screened, generated, ranked, and filtered through structural plausibility, developability, material compatibility, processing tolerance, and construct-readiness criteria before integration into smart biomaterials, hydrogel bioinks, scaffold surfaces, and three-dimensional bioprinted constructs. The final validation stage includes biochemical, cellular, material-level, biofabrication-level, and construct-level assessment. Experimental feedback, including assay results, material-performance data, cell responses, and construct-level outcomes, can support iterative model updating, re-ranking, and next-round candidate refinement.

For biomaterial and tissue-engineering applications, peptide suitability requires more than biological activity in solution. Candidate peptides must remain stable, soluble, safe, modifiable, and functional after chemical modification, immobilization, hydrogel incorporation, crosslinking, printing, and construct maturation (Kumar et al., 2025; Li G. et al., 2025). Foundation model-assisted workflows provide a complementary route for ranking, generating, and prioritizing candidates under these constraints (Goles et al., 2024; Liu et al., 2025c). However, computational prediction should be viewed as an early selection step rather than final validation. Figure 1 summarizes this transition from sequence-level candidate discovery to material-aware filtering, staged validation, and feedback-guided refinement.

## 3 Foundations of foundation models and multimodal modeling for material-aware peptide design

FMs reflect a broader shift in computational biology toward treating biological sequences as learnable information systems (Leclercq and Droit, 2026). In current peptide-design practice, this shift is most often implemented through PLM-based or sequence-level foundation model-assisted representations. Multimodal foundation model-enabled design should be reserved for workflows that integrate sequence information with additional modalities such as structure, physicochemical features, functional assays, material formulation, processing variables, imaging, or construct-level readouts (Elnaggar et al., 2022). For material-aware peptide design, these representations must ultimately be connected with molecular structure, physicochemical behavior, chemical modification, processing conditions, and construct-level function (Ross et al., 2022).

### 3.1 PLMs and sequence representation learning

PLMs adapt Transformer architectures such as Bidirectional Encoder Representations from Transformers (BERT) and Text-to-Text Transfer Transformer (T5) to amino acid sequences. Through self-supervised pretraining, they learn contextual relationships that can support peptide-specific prediction and generation tasks (Elnaggar et al., 2022; Leclercq and Droit, 2026). Representative model families include Evolutionary Scale Modeling (ESM) models such as ESM-1b and ESM-2, ProtBERT, ProtT5, and the autoregressive ProtGPT2 model (Rives et al., 2021; Elnaggar et al., 2022; Ferruz et al., 2022). Although their architectures and training objectives differ, they convert amino acid sequences into high-dimensional representations. These embeddings provide sequence-level information for predictive, generative, and multimodal models.

Compared with conventional descriptors such as charge, hydrophobicity, molecular weight, and amino acid composition, PLM embeddings capture broader sequence context. They may encode patterns associated with binding, folding, stability, activity, and functional motifs. Their utility, however, depends on the downstream task and the quality of the training labels. PLM embeddings are not direct measurements of thermodynamics, conformational dynamics, or material behavior. A model trained on soluble peptides may therefore provide limited information about function after conjugation, crosslinking, or hydrogel incorporation (Dai et al., 2025).

### 3.2 From sequence embeddings to peptide–target interaction and functional prediction

Once sequences are embedded, downstream models map peptide and target representations to binding probabilities, affinity-related scores, specificity, or activity. Representations may be combined through concatenation, similarity functions, bilinear layers, or cross-attention. Contrastive Language–Image Pre-training-like frameworks treat validated interacting pairs as positives and mismatched pairs as negatives, enabling large-scale ranking before experimental testing (Singh et al., 2023). Supervised classification and regression provide another route (Huang et al., 2024; Jia et al., 2024). Joint peptide–target representations can be processed by multilayer perceptrons, convolutional modules, attention layers, or ranking networks. Dual encoders support efficient screening of large candidate libraries, whereas cross-attention can model more detailed peptide–target relationships. The latter generally requires more computational resources and better aligned training data.

Predicted molecular compatibility does not necessarily translate into biological or material function. A peptide with favorable binding may lose activity after immobilization, hydrogel incorporation, crosslinking, printing, or prolonged culture. Biomaterial-related predictors must therefore distinguish molecular recognition from downstream functions such as adhesion, differentiation, antimicrobial activity, immune regulation, or matrix remodeling (Falcone et al., 2023). These models should be treated as prioritization tools rather than direct measures of biomaterial performance. Their reliability depends on the selected endpoint, assay conditions, and definitions of positive and negative examples. Verified non-binders are scarce, while random or shuffled negatives may create artificially simple tasks and inflate performance (Fernández-Díaz et al., 2024). Reliable evaluation therefore requires realistic negative sampling and external or experimental validation (Bizzotto et al., 2024).

### 3.3 Sequence-only, structure-aware, and multimodal modeling

Sequence-only models are scalable because they do not require solved peptide–target complexes or structural simulations for every candidate. They are therefore useful for early screening, retrieval, *de novo* generation, and targets with limited structural information. Their limitations become more apparent when function depends on flexible interfaces, transient conformations, or material-mediated spatial presentation (Xie et al., 2024). Sequence alone cannot capture all determinants of biomaterial performance. Binding and activity also depend on three-dimensional geometry, conformational compatibility, chemical modification, and motif accessibility (Slone et al., 2025). Material incorporation introduces further variables, including polymer composition, stiffness, degradation, crosslinking, shear stress, release behavior, and cell–matrix interactions (Xu et al., 2022). Structure-aware approaches address part of this gap by incorporating predicted or experimental structures, contact maps, graph representations, docking results, or conformational descriptors (Guan J. et al., 2025; Slone et al., 2025). However, adding structural information does not by itself make a model material-aware.

Multimodal peptide models can be considered at three levels. Molecular multimodal models combine sequence with structural or physicochemical descriptors. Interaction-aware models add target, geometric, or assay information. Material-aware multimodal models further incorporate formulation, conjugation chemistry, crosslinking conditions, rheology, printability, imaging, cellular responses, or construct-level outcomes (Bagherpour et al., 2025). Among these categories, material-aware models most closely align with multimodal foundation model-enabled design in the biomaterial context. These modalities may be integrated through feature concatenation, cross-attention, graph-based representations, or staged pipelines. Feature concatenation is simple and suitable for smaller datasets, but it may overlook complex interactions between modalities. Attention and graph-based methods can represent richer relationships, although they require better aligned data. Staged pipelines are more practical when datasets are heterogeneous, but uncertainty may accumulate across successive steps. Most current multimodal peptide models remain focused on molecular inputs rather than direct material-processing or construct-level data. Sequence-only, structure-aware, and material-aware approaches should therefore be viewed as complementary rather than interchangeable (Mondésert et al., 2025).

### 3.4 Interpretability goals in biomaterial peptide design

Interpretability should be defined by the experimental decisions that a model is expected to support. In biomaterial applications, key goals include identifying modification-tolerant sites, locating regions prone to inactivation, and assessing compatibility with crosslinking chemistry. Residue-level attribution may help identify sites for linker attachment or polymer conjugation while preserving functional motifs (Huang et al., 2024; Gao et al., 2025). Interpretation should also account for material context. A peptide may behave differently in solution and after immobilization because orientation, ligand density, motif accessibility, local retention, and release kinetics affect its apparent function. Models may help formulate hypotheses about these changes, but their outputs should not be treated as established mechanisms.

Attention scores and attribution maps require experimental confirmation through residue substitution, alanine scanning, site-specific conjugation, or comparison of free and immobilized peptides. Crosslinking compatibility remains particularly underdeveloped because few datasets directly connect peptide sequence with specific material reactions. Interpretability is therefore most useful when it guides testable experiments rather than providing only a visual explanation of model predictions (Bagherpour et al., 2025).

### 3.5 Data resources, multimodal labels, and material-context annotation challenges

The performance of FMs is ultimately constrained by dataset quality (Asim et al., 2025). Unlabeled sequence repositories are large, but labeled data for peptide interactions, activity, developability, material compatibility, and construct performance remain limited and poorly standardized (Meng, 2025). Sequence-level labels also rarely describe behavior after modification, scaffold conjugation, hydrogel incorporation, printing, or maturation (Perin et al., 2025). This creates a mismatch between rich pretrained representations and limited material-specific supervision. A model may encode detailed sequence information but lack the labels required to predict function after material integration. Heterogeneous assays, conditions, and reporting formats further limit transferability. Material-related outcomes are often measured using different formulations, time points, cell types, and processing conditions. Labels should therefore distinguish solution-phase activity, immobilized function, hydrogel performance, biofabrication tolerance, and construct-level outcomes. A single active–inactive label may obscure substantial context dependence.

Negative labels require similar caution. Synthetic negatives may be too easy, whereas presumed negatives may be context-dependent or simply untested (Sidorczuk et al., 2022). Closely related peptides or homologous targets may also appear in both training and test sets, leading to optimistic estimates (Fernández-Díaz et al., 2024). These issues reinforce the need for standardized annotation, transparent data provenance, and material-specific validation. Detailed benchmark design and validation criteria are considered separately in the evaluation framework. Material-aware FM design therefore depends on four connected foundations: transferable sequence representations, appropriate integration of molecular and material modalities, interpretable outputs linked to experimental decisions, and labels that reflect the intended material environment.

## 4 Foundation model-assisted peptide screening, generation, and material-aware optimization strategies

Current foundation model-assisted peptide design can be organized into retrieval and contrastive learning, generative modeling, discriminative prediction, preference optimization, and hybrid pipelines (Hashemi et al., 2024). These strategies differ in how they search sequence space, use training data, incorporate constraints, and connect predictions with experimental validation (Ekambaram and Dokholyan, 2026). In most current examples, the computational core remains PLM-based or sequence-level foundation model-assisted. The term “multimodal foundation model-enabled” is most appropriate when sequence, structural, functional, material, and experimental information are integrated within a shared workflow. A material-aware workflow may combine retrieval or generation with activity prediction, structural refinement, physicochemical filtering, and material-level evaluation (Ahmed et al., 2024). Its practical value depends not only on molecular prediction, but also on whether the selected peptide can tolerate modification, incorporation, processing, and validation in the final biomaterial system.

### 4.1 Retrieval and contrastive-learning frameworks for candidate enrichment

When large peptide libraries are already available, the challenge is often not generating additional sequences but identifying which candidates are most worthy of experimental follow-up. Retrieval and contrastive-learning frameworks address this need by organizing peptides and targets within a shared embedding space, where biologically compatible pairs are positioned closer together than unrelated combinations. Given a protein, receptor, matrix molecule, or functional goal, the trained model ranks existing candidates by predicted compatibility (Siwek et al., 2025).

These systems commonly encode peptides and targets with pretrained PLMs or task-specific encoders. Contrastive objectives reward validated pairs and penalize mismatches (Yan et al., 2025). Dual encoders are efficient because peptide and target embeddings can be calculated separately and compared across large libraries. As a result, retrieval-based strategies are well suited for front-end candidate enrichment before synthesis, scaffold functionalization, hydrogel conjugation, or cellular testing (Hargrove et al., 2026). Their value becomes especially apparent when the search space already contains display-derived peptides, natural fragments, motif collections, or synthetic libraries. Rather than expanding sequence space, retrieval methods focus on narrowing it, reducing the number of candidates that must undergo costly material-incorporation or functional assays. When material-context annotations are available, retrieval may also help prioritize peptides that are more likely to tolerate immobilization, crosslinking, polymer incorporation, or three-dimensional culture.

The principal limitation is that retrieval can only rank candidates that already exist within the indexed search space. Compared with generative models, retrieval methods are easier to trace and validate, but they are highly sensitive to the quality of positive and negative pairs (Cao et al., 2025). Their ability to support material-aware decision-making is also constrained when formulation variables, processing parameters, or construct-level annotations are unavailable.

### 4.2 Generative models for *de novo* peptide design

While retrieval-based approaches are effective when suitable candidates already exist, many biomaterial applications require functions that are poorly represented in current peptide collections. In such cases, generative models provide an alternative route by exploring previously uncharacterized regions of sequence space and proposing entirely new candidates (Chen et al., 2025; Nie et al., 2025). This capability is particularly valuable when known motifs cannot simultaneously satisfy requirements for activity, stability, safety, and material compatibility.

Generative peptide design can use several paradigms. Autoregressive models generate amino acid sequences step by step, whereas masked or denoising models can complete, edit, or repair sequence regions. Latent-space and diffusion-inspired methods explore continuous or structured representations of peptide space, and conditional generators attempt to bias sequence design toward predefined targets or properties (Wang Y. et al., 2025; Qian et al., 2026). Each paradigm offers distinct advantages. Autoregressive models are effective for generating complete sequences but may accumulate local errors during iterative prediction. Masked models are often better suited for motif completion or localized sequence modification. Latent-space and diffusion-based approaches can sample broader regions of sequence space, although their outputs typically require more stringent downstream filtering. Conditional generation becomes particularly attractive when design objectives extend beyond activity alone and incorporate developability or material-processing constraints.

A generated sequence may appear plausible from a modeling perspective while still failing during experimental development. Predicted activity does not guarantee stability, solubility, safety, synthetic accessibility, or compatibility with material-processing workflows. For biomaterials and hydrogel bioinks, additional considerations include modification tolerance, crosslinking compatibility, matrix retention, printability, and preservation of cellular function following material incorporation. Compared with retrieval frameworks, generative models offer substantially greater sequence-space exploration but also introduce a larger validation burden. At present, most peptide generators continue to be evaluated primarily using sequence validity, novelty, diversity, or predicted activity, whereas hydrogel compatibility, printing tolerance, and construct-level functionality are assessed far less frequently. Consequently, generated candidates generally require additional filtering through discriminative models, developability assessment, and experimental testing before being considered realistic material-design solutions (Ekambaram and Dokholyan, 2026).

### 4.3 Discriminative models for binding, property, and functional prediction

In practical peptide-design workflows, the central challenge is often not generating candidates but determining which candidates should be advanced and which should be discarded. Discriminative models are particularly valuable at this stage because they convert sequence information into specific experimentally relevant predictions (Badrinarayanan et al., 2025). Within foundation model-assisted peptide design, these predictions generally fall into three categories: bioactivity (e.g., binding, antimicrobial activity, or immunological recognition) (Xiong et al., 2024), developability and safety (e.g., stability, solubility, aggregation, hemolysis, or cytotoxicity), and material-related performance (e.g., modification tolerance, hydrogel compatibility, three-dimensional culture behavior, or functional retention after conjugation). One common application is peptide–target interaction prediction. Peptide and target representations are provided as input, and the model estimates the likelihood of interaction. PLM-derived embeddings provide a useful sequence-level foundation because they capture contextual sequence information that can be processed through classification heads, cross-attention mechanisms, or interaction networks (Siwek et al., 2025). In many workflows, these models serve as a second layer of prioritization following retrieval or generative design.

Discriminative models are also useful for excluding candidates with unfavorable properties. For biomaterial applications, this filtering process must extend beyond intrinsic molecular activity. A peptide may satisfy a binding or activity threshold but still fail because it aggregates, loses function after immobilization, interferes with crosslinking, or performs poorly in a hydrogel or scaffold context (Blomstrand et al., 2024). An advantage of discriminative approaches is that their predefined endpoints make benchmarking relatively straightforward (Cao et al., 2025). However, this strength also creates an important limitation. Model outputs are inherently restricted by the labels used during training. A predictor developed for toxicity, binding, or antimicrobial activity cannot automatically be expected to capture material retention, crosslinking tolerance, bioink processability, or construct-level functionality. Accordingly, discriminative models are most useful as endpoint-specific decision-support tools and should not be interpreted as replacements for material-based or biological validation.

### 4.4 Preference optimization and multi-objective material-aware design

Many early AI-guided peptide-design studies focused on optimizing a single objective, typically binding affinity, biological activity, or sequence plausibility (Liu et al., 2024). In practice, however, biomaterial and tissue-engineering applications rarely allow such a simplified optimization strategy (Wu et al., 2024). A useful peptide must simultaneously satisfy multiple requirements, including biological activity, stability, selectivity, solubility, synthetic feasibility, low cytotoxicity, material compatibility, spatial presentation, and performance within scaffold or hydrogel environments.

Preference-optimization approaches attempt to address this challenge by introducing an additional decision-making layer into peptide-design workflows. Instead of selecting candidates solely according to predicted activity, these methods rank peptides using preference functions that may integrate experimental rankings, predicted properties, expert knowledge, or application-specific constraints (Jiang et al., 2025). This distinction is important because the peptide with the highest predicted activity is not necessarily the peptide most likely to succeed within a biomaterial system.

A practical material-aware workflow should distinguish between hard constraints and soft objectives. Hard constraints define conditions under which a candidate is excluded, such as unacceptable toxicity, severe aggregation propensity, poor synthetic feasibility, loss of activity following immobilization, or incompatibility with required crosslinking chemistries. Soft objectives, by contrast, can be balanced through weighting, ranking, or multi-criteria optimization and may include binding strength, solubility, release characteristics, retention, stability, and differentiation-supporting properties.

Conflicts among objectives often complicate candidate selection. A peptide with excellent predicted binding performance may still be unsuitable if it exhibits poor material compatibility, even when a composite scoring system assigns it a favorable overall score. Under these circumstances, Pareto-front approaches or constraint-aware ranking frameworks may provide more informative decision support than a single weighted metric, particularly when activity, safety, developability, and material performance cannot be optimized simultaneously (Liu et al., 2025a).

Preference optimization is especially relevant for hydrogel bioinks and tissue-engineering constructs because peptide performance emerges from the interaction between molecular function and material context. Its effectiveness, however, remains dependent on the quality of available preference data. Rankings derived from one target, material system, or assay environment may not transfer reliably to different experimental settings without additional validation.

### 4.5 Hybrid pipelines linking sequence, structure, property, and material filters

No single computational model currently captures the full spectrum of constraints involved in biomaterial peptide design. For this reason, many contemporary workflows increasingly rely on hybrid strategies that integrate multiple modeling layers into a coordinated decision-making process. A typical pipeline may begin with retrieval, *de novo* generation, or mutation proposal, followed by structural modeling, docking or dynamics-based evaluation, graph-based analysis, physicochemical filtering, and material-aware assessment (Abramson et al., 2024; Chen et al., 2024; Wu et al., 2026).

The motivation for combining these approaches is straightforward. PLMs are highly effective for sequence exploration and early-stage prioritization, yet later stages of candidate selection often require additional information regarding binding geometry, conformational compatibility, developability, chemical modification, and material integration. By integrating complementary sources of information, hybrid pipelines can identify candidates that are not only statistically plausible but also mechanistically credible and experimentally actionable.

Within biomaterial applications, these workflows may be extended to evaluate modification strategies, conjugation chemistry, hydrogel compatibility, polymer–peptide interactions, printability, and construct-level performance (Geevarghese et al., 2025). Hybrid pipelines may also generate interpretable design insights, including candidate contact residues, modification-sensitive regions, linker-selection strategies, or experimental priorities. Such outputs are most valuable when they guide experimental planning and hypothesis generation rather than being treated as definitive evidence of function.

Despite their flexibility, hybrid pipelines inherit uncertainty from every modeling layer they contain. Structural predictions may be unreliable for highly flexible peptides or incompletely characterized targets, while docking and molecular-dynamics simulations remain sensitive to modeling assumptions. Material-aware predictions introduce additional complexity because performance is strongly influenced by polymer composition, stiffness, degradation behavior, crosslinking conditions, cell type, and culture environment. Consequently, the greatest strength of hybrid workflows lies in organizing candidate selection and reducing experimental search space (Zech et al., 2025). Their outputs should be interpreted as prioritization tools that support validation efforts, not as guarantees that a peptide is ready for biomaterial deployment.

### 4.6 Multimodal and closed-loop design-test-learn workflows

A recurring limitation of many computational peptide-design studies is that model predictions and experimental observations remain only loosely connected. Design–test–learn frameworks seek to address this disconnect by incorporating experimental outcomes into subsequent rounds of computational prioritization or optimization (Liu et al., 2025c). In this review, “closed-loop” is used cautiously: a complete closed-loop workflow would require automated candidate selection, experimental testing, data capture, model updating, and re-design within a defined material-development cycle. Most current examples instead demonstrate design–test–learn logic, partial automation, or feedback-informed optimization. Within these workflows, peptide candidates are evaluated using broader criteria than sequence plausibility or predicted binding alone, including structural feasibility, physicochemical suitability, material compatibility, processing tolerance, and construct-level biological performance (Chen et al., 2026).

A typical design-test-learn cycle begins with the definition of biological and material objectives. Candidate peptides are then retrieved, generated, or modified. Hard filters remove sequences associated with unacceptable toxicity, poor synthetic feasibility, severe aggregation, or predicted material incompatibility. The remaining candidates are prioritized according to predicted performance, uncertainty, and diversity. Experimental evaluation follows at solution, material, and construct levels, after which newly generated data are incorporated through retraining, fine-tuning, Bayesian updating, active learning, or reinforcement-learning strategies (Talluri et al., 2025). The cycle continues until performance improvements plateau or experimental resources become limiting.

Different feedback mechanisms contribute in different ways. Active learning preferentially selects candidates expected to provide the most informative data, often because they are uncertain, underrepresented, or highly diverse. Bayesian optimization seeks improved solutions under restricted experimental budgets (Torres et al., 2026). Fine-tuning and retraining update predictive models using newly acquired observations, whereas reinforcement-learning approaches can optimize sequence generation when an appropriate reward function is available. For biomaterial applications, these reward functions should reflect not only biological activity but also developability, material compatibility, processing tolerance, and construct-level performance.

Evidence from adjacent fields illustrates the value of this strategy without proving a complete closed-loop workflow for functional tissue-engineering materials. Experiment-driven machine-learning systems have identified previously unrecognized self-assembling peptide materials through iterative cycles of synthesis and testing (Talluri et al., 2025). Similarly, generative optimization has been applied successfully to antimicrobial peptide development with experimental validation, showing how computational exploration can be linked to functional improvement outside biomaterial applications (Torres et al., 2026). These studies should therefore be viewed as illustrative examples of design–test–learn logic and partially automated optimization, rather than complete closed-loop workflows for functional tissue-engineering materials.

By integrating material and biological feedback into optimization, design–test–learn frameworks may align model development with tissue-engineering objectives instead of relying solely on sequence-level metrics. Multi-source and multi-institutional datasets could eventually support more generalizable relationships among sequence, structure, processing conditions, material context, and biological function (Singh et al., 2026). At present, however, data remain sparse and heterogeneous, and fully automated closed-loop systems should be considered emerging research goals rather than mature design infrastructures.

**FIGURE 2:**
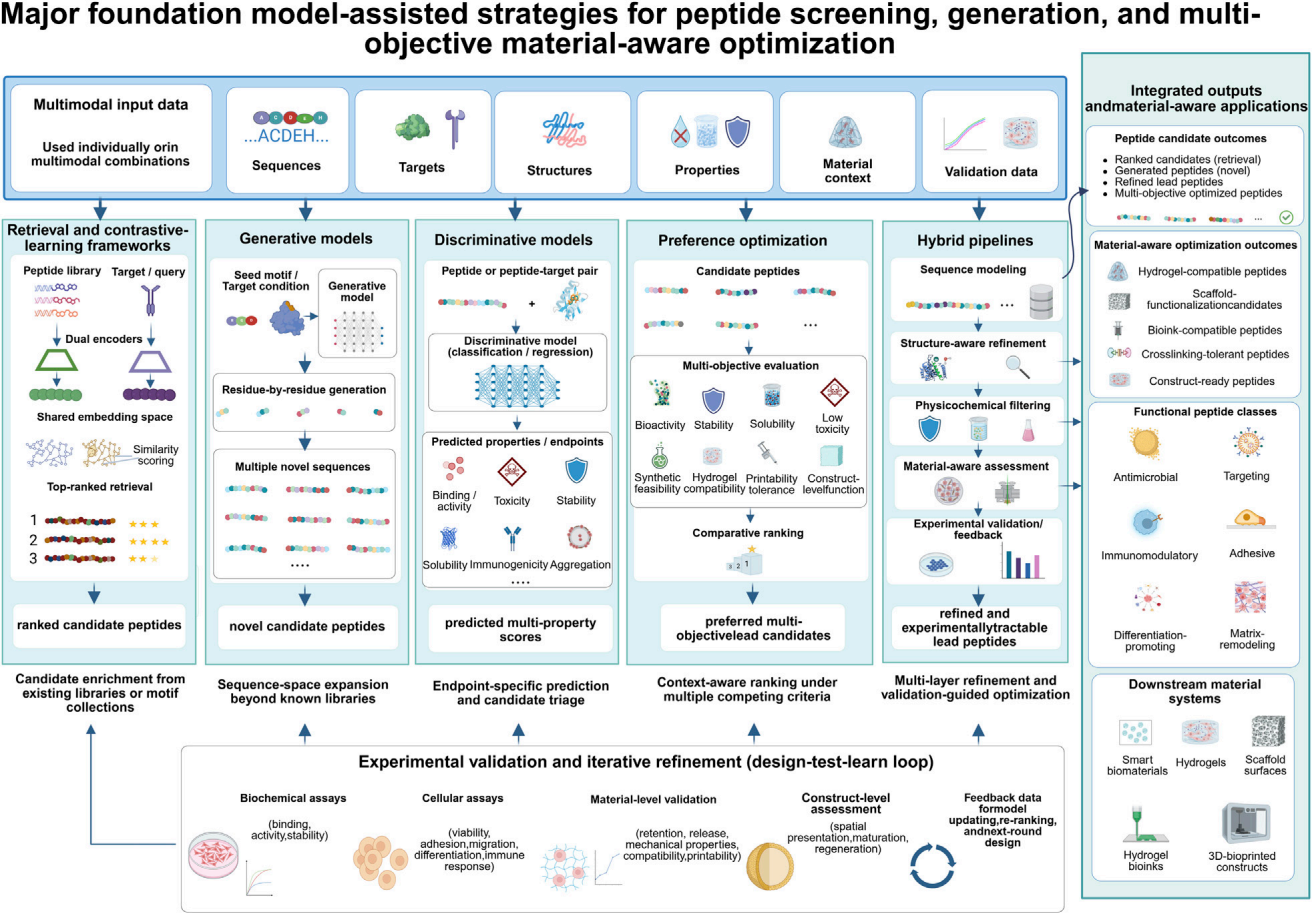
Major foundation model-assisted strategies for peptide screening, generation, and multi-objective material-aware optimization. The figure compares retrieval and contrastive-learning frameworks, generative models, discriminative models, preference optimization, and hybrid pipelines. These strategies differ in their input modalities, design objectives, outputs, validation requirements, and current maturity. Retrieval and discriminative prediction are closer to practical candidate enrichment and endpoint-specific triage, whereas material-aware generation, preference optimization, hybrid refinement, and closed-loop workflows still depend on stronger experimental feedback, standardized datasets, and material-level validation. The integrated output panel highlights peptide candidate outcomes, material-aware optimization outcomes, functional peptide classes, and downstream material systems. The lower design-test-learn loop emphasizes the need to connect biochemical assays, cellular assays, material-level validation, construct-level assessment, and model updating.

Taken together, foundation model-assisted peptide design is best understood as an integrated framework rather than a collection of isolated algorithms. Retrieval enriches existing candidate pools, generative models expand sequence space, discriminative predictors support targeted triage, preference optimization balances competing objectives, and hybrid workflows connect sequence-level modeling with structural and material-aware refinement. Design–test–learn systems add iterative experimental learning, but their ultimate reliability will depend on standardized material-context datasets and rigorous validation across solution, material, printing, and construct-level environments. Figure 2 provides a visual overview of the major foundation model-assisted strategies discussed in this section, showing how retrieval, generation, discriminative prediction, preference optimization, and hybrid pipelines can be integrated into material-aware peptide design workflows. To complement this visual framework, Table 1 compares these strategies in terms of their typical inputs, outputs, strengths, limitations, and relevance to material-aware peptide optimization.

**TABLE 1:** Representative foundation model-assisted peptide design strategies for material-aware peptide screening and optimization.

Strategy	Representative approach	Main inputs/data	Material-aware information	Evidence and openness	Best use and main caveat
Retrieval and contrastive-learning frameworks	Dual encoders, shared embedding space, similarity-based ranking (Siwek et al., 2025)	Peptide libraries, motif collections, display-derived candidates, peptide–target pairs	Usually absent; can be extended with material-context annotations	Mainly V0-V1; code or data availability varies	Efficient enrichment of existing candidates, but cannot explore outside the indexed pool
Generative models (Chen et al., 2025)	Autoregressive, masked, denoising, latent-space, or diffusion-based peptide generation	Large peptide or protein sequence datasets; optional task-specific fine-tuning	Mostly physicochemical or predicted-property constraints; direct hydrogel, printing, and construct labels remain rare	Mainly V0-V1; some models provide code or weights	Expands sequence space, but novelty does not ensure synthesis feasibility, safety, or material compatibility
Discriminative prediction models (Badrinarayanan et al., 2025)	Classification or regression models using PLM embeddings, descriptors, graph features, or multimodal features	Labeled datasets for activity, binding, toxicity, stability, solubility, aggregation, or immunogenicity	Usually endpoint-specific; material incorporation and processing variables are rarely included	Mainly V0-V1; many predictors provide web servers, code, or benchmark datasets	Useful for endpoint-specific triage, but strongly depends on label quality and cannot infer material performance alone
Preference optimization and multi-objective ranking	Weighted ranking, constraint-aware scoring, Pareto-front selection, or preference learning (Liu et al., 2025a)	Candidate sequences with predicted or experimental activity, safety, developability, and application-specific scores	Can include hydrogel compatibility, printability, or crosslinking tolerance if explicitly encoded	Mainly V0-V1; experimental validation depends on follow-up studies	Balances competing objectives, but composite scores can hide failure in essential constraints
Hybrid pipelines (Zech et al., 2025)	Coarse-to-fine workflows combining retrieval or generation with structure prediction, docking, molecular dynamics, and property filtering	Sequence, target, structure, physicochemical properties, predicted developability, and optional material descriptors	Can incorporate modification tolerance, conjugation feasibility, hydrogel compatibility, and biofabrication constraints	V0-V2 in most cases; higher validation requires material and construct assays	Improves prioritization through multi-layer filtering, but uncertainty accumulates across sequential steps
Closed-loop design-test-learn workflows (Liu S. et al., 2026)	Iterative candidate selection, synthesis, testing, feedback integration, model updating, active learning, Bayesian optimization, or reinforcement learning	Candidate sequences, model predictions, assay results, material-performance data, and biological readouts	Potentially strong if feedback includes formulation, processing, failed candidates, and construct-level outcomes	V1-V4 possible; complete autonomous workflows remain emerging	Uses experimental feedback to improve next-round design, but requires standardized metadata and repeated validation

## 5 Evaluation metrics, benchmarking, and material-level validation

Foundation model-assisted peptide design demands evaluation frameworks that extend beyond conventional prediction metrics. The key question is not simply whether a model assigns the correct label, but whether the candidates it prioritizes remain effective throughout the full development pathway. A peptide may achieve favorable computational scores yet fail after chemical modification, hydrogel incorporation, scaffold conjugation, bioink formulation, or three-dimensional (3D) bioprinting (Falcone et al., 2023; Geevarghese et al., 2025).

From a translational perspective, evaluation should progress through multiple layers of evidence rather than relying on a single benchmark score. A practical hierarchy includes computational prediction, candidate prioritization, generative quality assessment, structural plausibility, developability and safety evaluation, material integration, and construct-level biological function. This perspective is particularly relevant in smart biomaterials and tissue engineering, where the intended product is often a peptide-functionalized material rather than an isolated peptide molecule. Accordingly, benchmark performance should be regarded as an early indicator of potential utility rather than direct evidence of readiness for biomaterial deployment.

### 5.1 Evaluation beyond predictive performance

Predictive performance remains an important starting point for model assessment, but it captures only part of what determines success in material-aware peptide design. Favorable sequence-level performance does not necessarily translate into retained function after material integration and processing (Fernández-Díaz et al., 2024). Evaluation for biomaterial applications should therefore determine whether computational prioritization identifies candidates that retain material-level function rather than focusing solely on sequence-level benchmark performance.

Failures may emerge at multiple stages of this transition. A peptide that is active in solution can lose activity following conjugation (Blomstrand et al., 2024). Another candidate may retain target binding while aggregating during formulation or interfering with crosslinking reactions. In other cases, molecular activity is preserved but downstream outcomes such as cell viability, differentiation, or matrix remodeling remain unsatisfactory in three-dimensional culture systems. These examples illustrate why computational performance and experimental utility should not be treated as interchangeable concepts.

A more informative evaluation strategy is to accumulate evidence across progressively more realistic testing environments. Computational metrics can prioritize candidates, biochemical assays can assess soluble activity, material-level experiments can determine whether functionality is retained after incorporation, and construct-level studies can evaluate the intended biological outcome. This staged approach reduces the risk of equating favorable model outputs with successful biomaterial performance.

### 5.2 Classification, ranking, and prioritization metrics

Classification metrics remain widely used when models are trained to predict predefined peptide labels. Although accuracy is straightforward to interpret, it may provide a misleading picture when datasets are imbalanced (Bizzotto et al., 2024). Metrics such as F1 score, Matthews correlation coefficient, area under the receiver operating characteristic curve, and area under the precision–recall curve often provide a more informative assessment under these conditions (Ebrahimikondori et al., 2024). The meaning of these metrics depends heavily on how labels and negative examples are defined. Artificially generated negatives can simplify classification tasks, whereas presumed inactive peptides may simply reflect a lack of experimental testing rather than true inactivity (Sidorczuk et al., 2022; Fernández-Díaz et al., 2024). Material-aware peptide design introduces an additional complication: a peptide that loses activity after immobilization represents a fundamentally different outcome from a peptide that never possessed intrinsic activity in the first place.

In many practical settings, ranking performance is more informative than global classification accuracy because researchers typically select only a small number of candidates for experimental evaluation (Pandey et al., 2025). Metrics such as top-k hit rate, precision@k, recall@k, enrichment factor, and rank correlation with experimental measurements provide a more direct indication of whether a model can enrich promising candidates for synthesis, material incorporation, or biological testing. Composite prioritization scores can further integrate predicted activity, safety, developability, and material compatibility into a single ranking framework. However, the usefulness of such scores depends on transparent weighting schemes that reflect the intended application. Without subsequent material-level validation, composite scores remain decision-support tools that assist candidate selection rather than evidence of final biological or material performance.

### 5.3 Evaluation of generative and structure-aware models

Assessing generative peptide models presents challenges that differ fundamentally from those encountered in conventional classification tasks. Unlike predictive models that evaluate existing sequences, generative systems create entirely new candidates, making it necessary to evaluate not only prediction quality but also the characteristics of the generated sequence space itself. Common computational metrics include validity, novelty, diversity, uniqueness, property satisfaction, and sequence plausibility (Özçelik and Grisoni, 2025). These measures provide useful information regarding whether a model can generate structurally coherent and sufficiently diverse peptide candidates. However, strong performance on generative benchmarks does not automatically imply biological relevance, experimental tractability, or compatibility with biomaterial applications.

For this reason, functional utility should be examined independently from generative quality. Generated peptides should be evaluated for synthetic accessibility, solubility, aggregation propensity, proteolytic stability, toxicity, modification tolerance, crosslinking compatibility, hydrogel retention, bioink processability, and performance in relevant cellular or construct-level assays (Sharick et al., 2023). Novel sequences are valuable only when they can ultimately be manufactured, incorporated into material systems, and validated under realistic experimental conditions.

Structure-aware evaluation provides an additional layer of confidence during candidate selection. Metrics such as docking scores, predicted binding energies, root-mean-square deviation, conformational stability, motif exposure, and accessibility of conjugation sites may help identify structurally implausible candidates or prioritize sequences for downstream testing (Zhai S. et al., 2025). These analyses can strengthen mechanistic interpretation and guide experimental planning.

Nevertheless, structural predictions should be interpreted cautiously. Favorable docking poses or predicted structures indicate plausibility rather than proof of function. Experimental measurements remain essential for confirming binding behavior, material compatibility, and biological performance. A practical strategy in multimodal design is to examine consistency across evaluation layers. Candidates prioritized at the sequence level should remain credible after structural refinement, physicochemical assessment, material-compatibility analysis, and biological testing. Discrepancies among these layers are often informative because they may reveal context-dependent limitations that would remain hidden if evaluation were restricted to a single modality.

### 5.4 Material-level validation hierarchy

One of the most common reasons computational peptide discoveries fail to translate into biomaterial applications is that validation frequently stops at the molecular or solution level. Yet the environments encountered during material fabrication and tissue engineering differ substantially from those represented in most computational benchmarks. A peptide that performs well in solution may behave very differently after immobilization, encapsulation, conjugation, printing, or prolonged culture.

To organize validation across computational and material levels, we introduce a practical V0–V4 hierarchy covering successive stages from computational assessment to construct-level functional validation. This hierarchy is intended to facilitate structured evaluation rather than to serve as a formal field-wide standard. It consists of V0 computational validation, V1 solution-level validation, V2 immobilized or hydrogel-level validation, V3 biofabrication or printing-level validation, and V4 construct-level functional validation. V0 represents computational evidence obtained before experimental testing begins, including classification performance, ranking quality, generative assessment, structural evaluation, physicochemical filtering, and uncertainty estimation. V1 focuses on soluble activity, such as binding affinity, potency, antimicrobial activity, serum stability, solubility, hemolysis, and cytotoxicity. Positive V1 results provide important molecular evidence but offer limited information about performance after material incorporation. V2 evaluates whether peptide function is retained after conjugation, adsorption, encapsulation, or matrix incorporation, using endpoints such as peptide retention, release behavior, ligand accessibility, hydrogel compatibility, local concentration, and cell responses within the material environment (Sharick et al., 2023). V3 extends evaluation to fabrication and processing conditions, including shear stress, crosslinking reactions, light exposure, extrusion forces, prolonged culture, rheological compatibility, printability, crosslinking tolerance, post-print activity, shape fidelity, and cell viability following fabrication (Geevarghese et al., 2025). V4 provides the strongest evidence by assessing whether the peptide-functionalized material achieves its intended biological purpose, such as adhesion, differentiation, extracellular-matrix deposition, tissue maturation, immune modulation, antimicrobial protection, vascularization, or other tissue-specific functions (Bagherpour et al., 2025). Viewed collectively, this hierarchy links computational prediction to translational outcomes through progressively more realistic testing environments. The framework does not eliminate uncertainty, but it provides a structured approach for determining where candidate failure occurs and for preventing early-stage computational success from being mistaken for biomaterial readiness.

### 5.5 Benchmark design, uncertainty, and reporting requirements

Reported model performance is influenced as much by benchmark design as by model architecture itself. Consequently, benchmark results should always be interpreted in the context of how datasets were constructed, partitioned, and evaluated. In peptide-design studies, random train–test splits often provide an optimistic estimate of performance because closely related peptides, homologous targets, or highly similar scaffolds may appear in both training and testing subsets. Under these conditions, models may benefit from memorizing local sequence patterns rather than learning relationships that generalize to genuinely novel candidates.

More informative benchmark strategies attempt to reduce this source of information leakage. Examples include sequence-similarity splits, target-based splits, scaffold-based splits, material-system splits, laboratory-specific splits, and time-based validation schemes (Bizzotto et al., 2024). The most appropriate benchmark is not necessarily the most difficult one, but the one that best reflects the intended deployment scenario. Dataset construction introduces additional sources of bias. Negative sampling is particularly influential because the definition of a negative example can substantially alter apparent model performance. Randomly generated negatives are often easy to separate from positive examples, whereas experimentally verified non-binders, difficult decoys, or context-specific negatives present a more realistic challenge (Sidorczuk et al., 2022). Material-aware peptide design introduces further complexity because failure may occur at multiple stages. Loss of activity after immobilization, incompatibility with printing processes, and complete absence of intrinsic activity represent distinct biological outcomes and should not be collapsed into a single negative category.

Another critical question is whether benchmark performance remains stable outside the dataset on which it was originally reported. Internal cross-validation is useful for model development, yet it provides only limited evidence regarding real-world applicability. Independent datasets, alternative laboratories, different hydrogel formulations, distinct cell systems, separate fabrication platforms, and prospective experimental studies offer substantially stronger evidence of generalizability (Fernández-Díaz et al., 2024). Models intended for biomaterial applications should ideally demonstrate robustness across at least some of these dimensions.

Uncertainty estimation and calibration are equally important but remain underreported in many peptide-design studies. This issue becomes particularly relevant when experimental validation is expensive and only a small number of candidates can be tested. Overconfident predictions may encourage unnecessary investment in weak candidates, whereas calibrated uncertainty estimates can support active-learning workflows, identify out-of-distribution sequences, and guide exploration of underrepresented regions of peptide space. For this reason, confidence estimates should be regarded as an integral component of model outputs rather than optional supplementary information whenever such estimates are available.

An important distinction should also be made between benchmark success and design utility. High benchmark scores do not guarantee practical value if the resulting candidates are unstable, difficult to synthesize, toxic, incompatible with material systems, or never evaluated under relevant experimental conditions. Conversely, a model with only moderate global performance may still be highly useful if it consistently enriches experimentally tractable candidates within a specific biomaterial context. Practical utility is ultimately determined by the quality of selected candidates rather than by benchmark metrics alone.

Transparent reporting is therefore essential for meaningful comparison across studies. Evaluation reports should clearly describe the prediction task, dataset origin, label definitions, negative-sampling procedures, partitioning strategy, baseline methods, uncertainty estimates, validation stage, and material context (Collins et al., 2024). Such information allows readers to determine whether reported performance reflects purely computational evaluation or has been supported by solution-level, material-level, biofabrication-level, or construct-level validation. Greater reporting transparency will improve reproducibility, facilitate comparison among studies, and help distinguish genuine translational progress from benchmark-specific optimization. Table 2 summarizes the major evaluation dimensions that should be considered when assessing foundation model-assisted peptide design, especially when candidates are intended for material incorporation, biofabrication, and construct-level functional validation.

**TABLE 2:** Evaluation hierarchy, benchmarking considerations, and material-level validation endpoints for foundation model-assisted peptide design.

Validation level	Evaluation focus	Representative metrics/endpoints	Main purpose	Key limitation	Material-aware extension	Recommended validation focus
V0 Computational validation (Bizzotto et al., 2024; Fernández-Díaz et al., 2024)	Model performance before experiments	Accuracy; F1 score; MCC; AUROC; AUPRC; precision@k; enrichment factor; novelty; diversity; docking score; RMSD; uncertainty or calibration	Candidate triage and benchmark comparison	Cannot prove experimental utility or material compatibility	Add material-context labels, material-aware negative examples, and validation-level annotation	Use appropriate baselines, non-random splits, hard negatives, uncertainty estimates, and external benchmarks
V1 Solution-level validation (Liu et al., 2025b)	Soluble activity and developability	Binding affinity; potency; antimicrobial activity; serum stability; solubility; aggregation; hemolysis; cytotoxicity	Confirm that predicted candidates show measurable activity or acceptable safety in solution	Does not test whether function is preserved after modification or material incorporation	Compare free, modified, and linker-bearing peptides	Use binding, activity, stability, and safety assays before material integration
V2 Immobilized or hydrogel-level validation (Sharick et al., 2023)	Function after conjugation, adsorption, encapsulation, or hydrogel incorporation	Peptide retention; release behavior; ligand accessibility; local concentration; hydrogel compatibility; cell response within the matrix	Determine whether peptide function is preserved in a material environment	Static hydrogel or scaffold assays may not capture printing or long-term construct behavior	Report conjugation chemistry, linker, ligand density, orientation, matrix composition, and degradation behavior	Validate free versus immobilized or hydrogel-incorporated peptide performance
V3 Biofabrication or printing-level validation (Geevarghese et al., 2025)	Processing tolerance and post-processing function	Rheology; printability; crosslinking tolerance; shape fidelity; post-print peptide activity; post-print cell viability	Assess whether peptide-functionalized materials tolerate fabrication conditions	Printing compatibility does not prove long-term biological function	Report nozzle, pressure, speed, temperature, crosslinking mode, light dose, enzyme concentration, and maturation conditions	Test peptide activity and cell response before and after extrusion, crosslinking, or printing
V4 Construct-level functional validation (Singh et al., 2026)	Final biological function in three-dimensional constructs or tissue-engineering models	Cell adhesion; migration; differentiation; matrix deposition; tissue maturation; immune modulation; antimicrobial protection; angiogenesis; tissue-specific function	Establish whether the peptide-functionalized material supports the intended biological outcome	Context-specific, slower, and harder to standardize across laboratories	Link peptide design with construct architecture, cell type, culture duration, matrix remodeling, and tissue-specific readouts	Use application-specific three-dimensional culture, organoid, infection, immune, vascularization, or regeneration models

MCC, matthews correlation coefficient; AUROC, area under the receiver operating characteristic curve; AUPRC, area under the precision-recall curve; RMSD, root-mean-square deviation; V0, computational validation; V1, solution-level validation; V2, immobilized or hydrogel-level validation; V3, biofabrication or printing-level validation; V4, construct-level functional validation Note: The V0–V4 hierarchy is used here as a practical organizing framework rather than as a formal field-wide standard.

## 6 Developability, material compatibility, and biofabrication tolerance

### 6.1 Limitations of predicted activity as a selection criterion

Predicted binding or biological activity does not by itself determine whether a peptide is suitable for biomaterial use. Candidate peptides must also remain stable, soluble, safe, manufacturable, and compatible with material processing (Liu et al., 2025b). Developability, material compatibility, and biofabrication tolerance should therefore be assessed before candidates are advanced to costly material-level or construct-level testing.

### 6.2 Key developability and material-compatibility dimensions

Key developability considerations can be divided into intrinsic peptide properties and material-dependent behaviors. Intrinsic peptide properties include proteolytic stability, solubility, aggregation tendency, synthetic feasibility, manufacturability, and safety-related characteristics such as hemolysis and cytotoxicity.

Proteolytic stability is essential because peptide-functionalized materials often operate in enzyme-rich and cell-remodeling environments. Peptides incorporated into hydrogels, scaffolds, or tissue-engineering constructs may encounter serum proteins, proteases, inflammatory cells, and matrix-remodeling processes. Rapid degradation can shorten the functional lifetime of a peptide, even when the initial activity is strong (Ross et al., 2022). Solubility and aggregation also influence practical utility. Poorly soluble or aggregation-prone peptides are difficult to formulate, conjugate, distribute uniformly, or retain reproducibly within hydrogel networks and printed bioinks. Aggregation may also alter local presentation and reduce biological consistency. These properties should be considered before formulation and conjugation rather than treated only as late-stage failures (Oeller et al., 2023). Safety-related properties remain central to candidate selection. Cytotoxicity, hemolysis, immunogenicity, and off-target membrane disruption can limit otherwise active peptides (Ebrahimikondori et al., 2024; Zhang S. et al., 2025). This is particularly relevant for highly cationic or amphipathic sequences, including some antimicrobial or cell-penetrating peptides. For tissue-engineering applications, safety should be interpreted in the context of the intended cells, material environment, and culture duration.

Material-dependent behavior should be evaluated separately from peptide-intrinsic quality. It includes ligand orientation, ligand density, linker design, immobilization chemistry, crosslinking tolerance, spatial presentation, release behavior, and accessibility after hydrogel incorporation or scaffold conjugation. These features depend on the material environment, hydrogel network, scaffold chemistry, crosslinking method, printing conditions, and three-dimensional culture context (Pan et al., 2024). A peptide with favorable intrinsic properties may therefore perform poorly in a material if its functional motif is inaccessible, its release profile is unsuitable, or its activity is reduced by immobilization, crosslinking, or processing (Sharick et al., 2023; Xing et al., 2024). Manufacturability should also be considered early. Sequence length, residue composition, chemical liabilities, stereochemical complexity, required modifications, purification difficulty, and batch-to-batch reproducibility all affect practical development (Li Q. et al., 2025). A peptide that is difficult to synthesize, purify, store, or reproduce may have limited value even if it scores well computationally.

### 6.3 Candidate-level constraints in foundation model-assisted workflows

These candidate-level properties can be incorporated into foundation model-assisted workflows as hard filters, ranking factors, or design constraints (Badrinarayanan et al., 2025). Hard filters may exclude peptides with high predicted toxicity, severe aggregation risk, poor synthetic feasibility, or incompatible modification sites. Softer ranking factors may prioritize candidates with balanced activity, stability, solubility, safety, and material compatibility (Li Q. et al., 2025). The purpose of these constraints is not to replace experimental testing, but to reduce avoidable failures before material incorporation. For biomaterial applications, this means that a candidate should be judged not only by predicted molecular activity, but also by whether it can tolerate modification, conjugation, crosslinking, processing, and presentation within the intended material system (Sharick et al., 2023). A practical foundation model-assisted workflow should therefore treat developability and material compatibility as candidate-selection criteria rather than as afterthoughts.

## 7 Application domains in smart biomaterials, bioinks, and functional tissue engineering

Foundation model-assisted peptide design is still emerging, and the application areas discussed below are not equally mature. Antimicrobial peptide discovery and hydrogel or wound-material integration are supported by relatively direct examples, whereas targeting, immunomodulatory, adhesive, matrix-remodeling, and differentiation-regulating peptides remain more prospective in the context of foundation model-assisted biomaterial design. In these settings, peptides serve as functional modules for antimicrobial activity, targeting, immune regulation, adhesion, differentiation, matrix remodeling, and cell–matrix signaling in hydrogels, scaffolds, bioinks, and 3D-bioprinted constructs (Nun and Joy, 2023; López-Gómez et al., 2025). Their practical value depends on retaining function after modification, material incorporation, crosslinking, printing, and maturation (Wu et al., 2025; Netti et al., 2026).

### 7.1 Antimicrobial peptides for infection-resistant biomaterials

Applications are relatively mature among the areas discussed here because several direct examples of computational discovery and subsequent integration into hydrogels or wound materials are available. Their comparatively well-characterized relationships among charge, amphipathicity, hydrophobicity, and activity also facilitate model development (Brizuela et al., 2025). PLM-based and sequence-level foundation model-assisted methods can support sequence generation, activity prediction, toxicity screening, and multi-objective optimization (Bae et al., 2025; Guan C. et al., 2025). This is important because potent antimicrobial peptides may also cause hemolysis or cytotoxicity, show poor serum stability, or lose activity after immobilization.

Antimicrobial peptides can be incorporated into hydrogels, wound dressings, scaffold surfaces, and bioinks to reduce infection risk while supporting tissue repair (Wang H. et al., 2025). Evaluation should extend beyond minimum inhibitory concentration, hemolysis, and cytotoxicity to include retention, release, hydrogel compatibility, host-cell viability, and antimicrobial activity after crosslinking (Petit et al., 2025; Shahrour et al., 2025). Material-aware models may further integrate sequence features with membrane-disruption potential, degradation, hydrogel retention, and release kinetics. Such integration may help balance antimicrobial potency with safety and regenerative compatibility (Brizuela et al., 2025; Zervou et al., 2025; Liu Q. et al., 2026).

### 7.2 Targeting peptides for scaffold and construct functionalization

Targeting and tissue-homing peptides recognize specific cells, extracellular matrix components, vascular structures, or disease-associated environments (Gouveia et al., 2025; Rehman et al., 2025). In biomaterial contexts, these applications remain less mature than antimicrobial peptide design. Foundation model-assisted retrieval, discriminative prediction, and generation may identify short ligands from large sequence spaces, particularly when known motifs or labeled data are limited (Kong et al., 2025; Cho et al., 2026). These peptides can functionalize scaffold surfaces, nanoparticles, hydrogel networks, and 3D-bioprinted constructs (Gisbert-Garzarán et al., 2023; Omidian et al., 2025). Their performance depends on specificity, conjugation stability, orientation, linker design, and spatial presentation rather than affinity alone (Wu et al., 2025; Zhou and Ma, 2026). Material-aware workflows should therefore combine target recognition with conjugation-site selection, scaffold chemistry, and cell-specific responses, especially in vascularized or tissue-homing constructs (Nan et al., 2024; Guo et al., 2025).

### 7.3 Immunomodulatory peptides for regenerative microenvironments

Sequence-based models have supported immune-related tasks such as peptide–human leukocyte antigen binding, T-cell receptor recognition, neoantigen prioritization, and peptide vaccine design (Wang et al., 2024; Wang Z. et al., 2025; Vo et al., 2025). Although developed mainly for cancer immunotherapy, these methods provide a conceptual route for regenerative biomaterials because inflammation and host–material interactions shape tissue repair (Gomes et al., 2025; Salehi Moghaddam et al., 2025). In biomaterial systems, however, immunomodulatory peptide design remains prospective and requires validation in the relevant material and cellular context. Foundation model-assisted methods may prioritize peptides that regulate immune-cell recruitment, cytokine signaling, macrophage polarization, or tissue repair (Lu et al., 2025). Validation should include cytokine responses, macrophage phenotypes, cell compatibility, and construct maturation under the intended release or immobilization conditions (Li Z. et al., 2025; Zhai M. et al., 2025).

### 7.4 Adhesive, matrix-remodeling, and differentiation-regulating peptides for hydrogel bioinks and 3D-bioprinted constructs

Adhesive, matrix-remodeling, and differentiation-regulating peptides provide related but distinct cell-instructive cues within otherwise inert or weakly bioactive materials. Adhesive peptides should be validated mainly through cell attachment, spreading, focal adhesion formation, or integrin-related assays. Matrix-remodeling peptides require endpoints such as extracellular matrix deposition, degradation, remodeling-enzyme activity, matrix organization, or mechanical remodeling. Differentiation-regulating peptides require lineage-specific markers, gene or protein expression, functional maturation, or phenotype-specific readouts. In hydrogel bioinks and 3D-bioprinted constructs, these peptides may be conjugated to polymer networks, displayed on scaffold surfaces, or released in a controlled manner (Nizam et al., 2024; Sung et al., 2026). Their effects depend on ligand density, accessibility, spatial distribution, matrix stiffness, degradation, and tolerance to printing and crosslinking (Moghaddam et al., 2025).

Foundation model-assisted workflows may rank or generate candidates according to adhesion potential, receptor engagement, cytotoxicity, modification tolerance, hydrogel compatibility, and post-print cell function (Hao et al., 2023; Hang et al., 2025). Multimodal models can also incorporate microscopy, gene expression, cell morphology, matrix-remodeling indicators, and tissue-specific functional markers during iterative design-test-learn cycles (Ng et al., 2026). The goal is not merely to identify an active peptide, but to create a peptide-functionalized environment that directs cell behavior and supports functional tissue formation.

**FIGURE 3:**
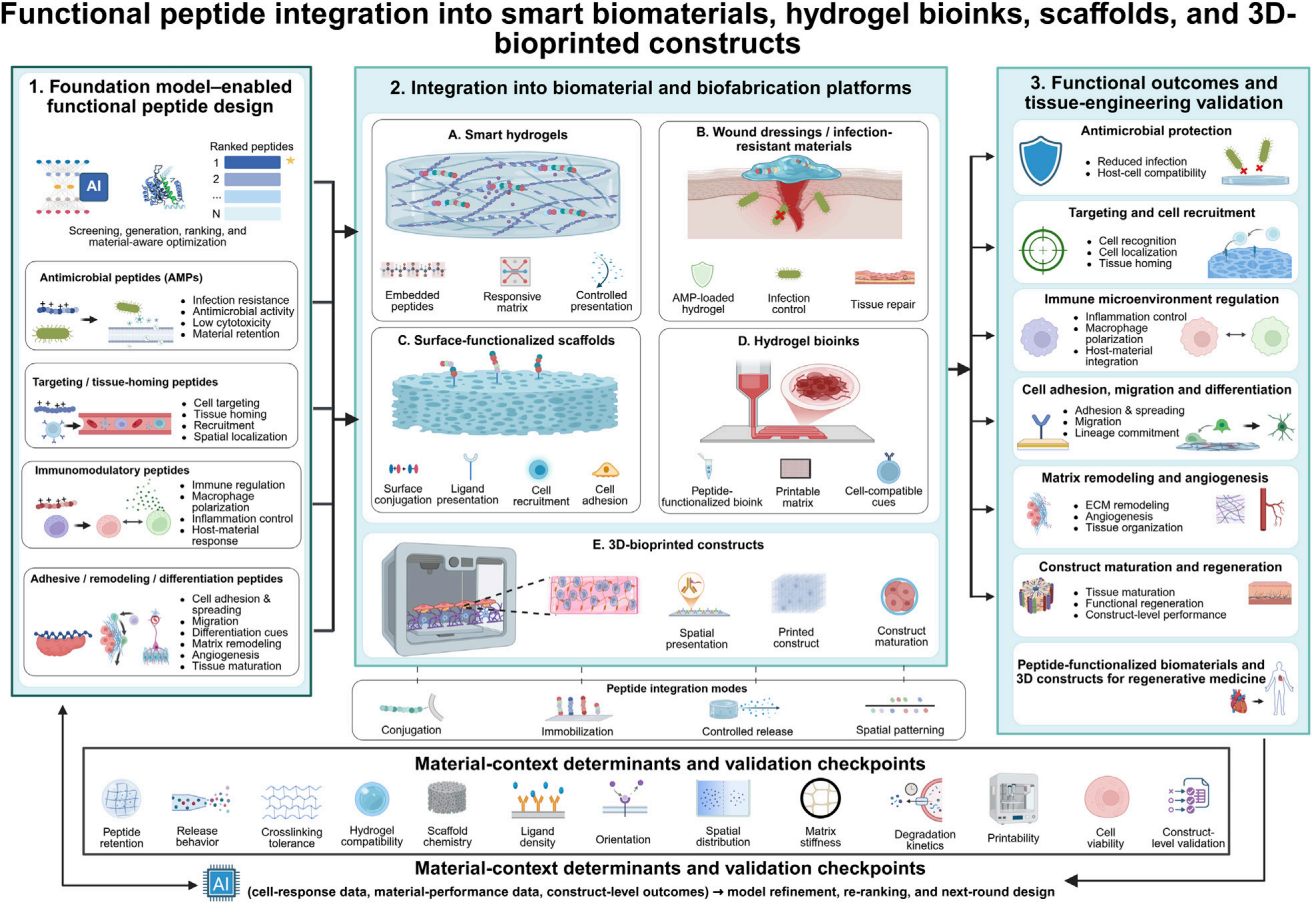
Functional peptide integration into smart biomaterials, hydrogel bioinks, scaffolds, and three-dimensional bioprinted constructs. The figure summarizes how foundation model-assisted peptide design can support the screening, generation, ranking, and prioritization of antimicrobial, targeting and tissue-homing, immunomodulatory, adhesive, matrix-remodeling, and differentiation-regulating peptides for biomaterial and biofabrication applications. These peptides can be incorporated into smart hydrogels, wound dressings and infection-resistant materials, surface-functionalized scaffolds, hydrogel bioinks, and three-dimensional bioprinted constructs through conjugation, immobilization, controlled release, or spatial patterning. Their integration may support antimicrobial protection, targeted cell recruitment, immune microenvironment regulation, cell adhesion, migration and differentiation, matrix remodeling and angiogenesis, and construct maturation and regeneration. Peptide function should be evaluated after chemical modification, material incorporation, crosslinking, printing, and construct maturation. Key material-context determinants and validation checkpoints include peptide retention, release behavior, crosslinking tolerance, hydrogel compatibility, scaffold chemistry, ligand density, orientation, spatial distribution, matrix stiffness, degradation kinetics, printability, cell viability, and construct-level functional validation. Experimental data generated at the material and construct levels can be fed back into model refinement, candidate re-ranking, and subsequent rounds of peptide design.

Overall, foundation model-assisted design may support infection-resistant materials, targeted constructs, regenerative immune modulation, and cell-instructive bioinks. Its value depends on connecting sequence design with material integration, biofabrication compatibility, and functional validation. Figure 3 illustrates how different classes of functional peptides can be integrated into smart biomaterials, hydrogel bioinks, scaffold systems, and 3D-bioprinted constructs to support antimicrobial protection, targeting, immune regulation, cell–matrix signaling, matrix remodeling, and construct maturation.

## 8 Discussion and future directions

### 8.1 Current bottlenecks and actionable solutions

Material-aware foundation model-assisted peptide design has advanced rapidly, but its use in smart biomaterials and functional tissue engineering remains constrained by data, validation, and translation barriers. Current models generally perform more consistently on sequence-level or target-level tasks than on material-related prediction tasks. The central challenge is whether models can support experimentally tractable candidates that retain function after modification, material incorporation, processing, and construct maturation.

Data scarcity remains a major bottleneck. Peptide datasets are often large at the sequence level but sparse in material-context labels, especially for conjugation, hydrogel incorporation, crosslinking, printability, and construct-level outcomes. A practical direction is to build multi-source datasets that connect sequence, structure, physicochemical properties, material formulation, processing conditions, imaging, and biological readouts (Basu et al., 2022; Konshin et al., 2025). These datasets should include both successful and failed candidates, because failure modes can reveal sequence liabilities, unsuitable modifications, or incompatible material environments.

Benchmark fragmentation also limits comparability. Different studies use different splits, label definitions, negative sampling strategies, and validation endpoints (Bizzotto et al., 2024). Future benchmarks should report the split strategy, negative-sampling logic, assay context, material system, and validation level (Fernández-Díaz et al., 2024). The aim should be to test generalization across new sequences, targets, scaffolds, material systems, and laboratories rather than to optimize performance on easy internal splits.

Interpretability remains underdeveloped for material-centered peptide design. Residue-level explanations are useful only when they support testable design decisions, such as where a peptide can be modified, which regions are prone to inactivation, and whether a sequence is compatible with a given crosslinking or immobilization strategy. These explanations should be validated through residue substitution, site-specific conjugation, and material-level testing.

Generative models create another challenge because novelty does not ensure synthesis feasibility, safety, stability, or material compatibility. Generated peptides should therefore be filtered through developability, toxicity, modification, crosslinking, and processing constraints before being treated as candidates for biomaterials (Özçelik and Grisoni, 2025). External validation is also essential. Prospective testing in predefined material systems, comparison with simpler baselines, and cross-laboratory evaluation will be needed to determine whether foundation model-assisted workflows provide practical value beyond conventional screening or empirical optimization.

### 8.2 Opportunities for material-aware multimodal FMs

The next stage of material-aware FM design is not simply adding more modalities. The more important task is to align each modality with experimentally meaningful material endpoints. Sequence and structure can support molecular plausibility, while material composition, processing variables, imaging, and cellular readouts can connect peptide design with functional performance in hydrogels, scaffolds, bioinks, and printed constructs.

Material-aware multimodal learning may help estimate whether peptide function is retained after conjugation, hydrogel incorporation, crosslinking, or printing. Such models should distinguish molecular compatibility from material compatibility. A peptide may bind its target in solution but fail after immobilization because of altered orientation, low accessibility, weak retention, or interference with the surrounding matrix. Future models should therefore learn relationships among peptide features, material chemistry, fabrication history, and biological outcome rather than treating peptide activity as an intrinsic sequence property alone (Seep et al., 2024). Retrieval-augmented design is another promising direction. Retrieval can ground generated candidates in known motifs, assay evidence, and material-compatible examples, thereby improving traceability and reducing unrealistic generation (Wei et al., 2025). This approach may be especially useful when direct training data are limited, because retrieved evidence can help users inspect whether a proposed sequence resembles previously validated peptides or falls outside known design space (Dong et al., 2026). Structure-token and geometry-aware methods may further connect sequence plausibility with functional realism (Yang et al., 2025). For biomaterials, geometry should include not only the peptide–target interface but also linker orientation, motif exposure, immobilization geometry, and spatial presentation within a matrix (Klabukov et al., 2025). These features may help assess whether a functional motif remains accessible after conjugation or hydrogel incorporation.

Closed-loop learning is valuable because it converts experimental outcomes into model-improving feedback. The most useful closed-loop systems will not only test successful candidates, but also record why failed candidates failed. This allows failures in synthesis, toxicity, immobilization, printability, or construct function to become training signals rather than discarded observations. Experiment-driven machine learning in peptide materials and generative peptide optimization in related biomedical settings illustrate how computational prioritization can be coupled with experimental feedback, although fully autonomous biomaterial peptide laboratories remain emerging (Talluri et al., 2025; Torres et al., 2026). Human oversight remains essential. Expert judgment should focus on decisions that are poorly captured by current datasets, including synthesis feasibility, material chemistry, fabrication practicality, biological relevance, and construct-level function (Izadifar et al., 2025). The most useful systems are likely to combine model-driven exploration with transparent human-in-the-loop decisions, especially when candidate peptides must be integrated into complex biomaterial or biofabrication workflows (Nahal et al., 2024).

### 8.3 Standardized multi-source datasets and experimental feedback

Progress in material-aware foundation model-assisted peptide design will depend on how experimental information is recorded. A minimum metadata standard is needed so that peptide, material, processing, and biological data can be reused across studies. Without these contextual variables, models may incorrectly treat peptide activity as a fixed sequence property and overlook the influence of material environment and processing history.

Minimum reporting should cover peptide identity, chemistry, target and assay context, material formulation, conjugation and presentation, processing conditions, biological system, validation outcome, data provenance, and model reporting. These categories should be reported in structured formats whenever possible, because free-text descriptions are difficult to reuse for benchmarking or closed-loop model updating. Table 3 converts the discussion of data scarcity into a practical reporting checklist. It is intended to support data reuse, benchmark construction, and design-test-learn workflows.

**TABLE 3:** Recommended metadata and reporting checklist for material-aware foundation model-assisted peptide design and validation.

Metadata category	Required items	Purpose	Recommended reporting format	Related validation level
Peptide identity (Seep et al., 2024)	Sequence; length; sequence version; D/L configuration; terminal modifications	Defines the candidate being modeled, synthesized, or tested	FASTA file or structured sequence field	V0–V4
Chemical modification and synthesis (Li Q. et al., 2025)	Modification type; linker; purity; synthesis route; purification method; storage condition	Affects stability, activity, toxicity, and reproducibility	Controlled fields plus protocol link or supplementary method	V1–V4
Target and assay context (Bizzotto et al., 2024; Fernández-Díaz et al., 2024)	Target; receptor; assay type; endpoint definition; unit; time point; positive and negative label definition	Prevents ambiguous labels and invalid comparison across datasets	Structured endpoint table with units and thresholds	V0–V4
Material formulation (Geevarghese et al., 2025)	Polymer type; polymer concentration; additives; stiffness; porosity; degradation behavior; swelling behavior	Defines the matrix environment that can change peptide accessibility and function	Material composition table with measured properties	V2–V4
Conjugation and presentation (Sharick et al., 2023)	Conjugation chemistry; immobilization mode; linker length; ligand density; orientation; retention or release profile	Controls motif exposure, local concentration, and functional retention	Chemistry and presentation fields with quantitative density when available	V2–V4
Processing and biofabrication (Resende et al., 2025)	Printing pressure; speed; nozzle size; temperature; crosslinking condition; light dose; enzyme concentration; maturation condition	Determines processing tolerance, printability, and post-processing activity	Fabrication protocol table with machine and parameter settings	V3–V4
Biological system (Hua and Gaharwar, 2026)	Cell type; passage; seeding density; medium; culture duration; co-culture condition; immune, infection, or tissue model	Defines biological context and endpoint relevance	Cell and assay metadata fields	V1–V4
Validation outcome (Fernández-Díaz et al., 2024)	Computational readout; Solution-level readout; hydrogel-level readout; printing-level readout; construct-level readout; failure mode	Clarifies evidence strength and material relevance	Validation-level label plus quantitative readout	V0–V4
Data provenance and quality control (Clayton et al., 2025)	Laboratory; batch; instrument; preprocessing; missing data; outliers; negative results; failed candidates	Supports reproducibility, bias assessment, and reuse for model training	Provenance record and quality-control checklist	V0–V4
Model reporting (Park et al., 2024)	Model version; training data source; feature representation; modality type; fusion strategy; split method; baseline model; uncertainty estimate	Supports model comparison, interpretation, and reproducibility	Model card or standardized reporting checklist	V0

V0, computational validation; V1, solution-level validation; V2, immobilized or hydrogel-level validation; V3, biofabrication or printing-level validation; V4, construct-level functional validation.

Experimental feedback should be treated as structured training data rather than isolated confirmation. Each tested candidate should ideally be linked to its sequence, modification state, material formulation, processing history, validation level, and failure mode. Failed candidates should be recorded with specific labels, such as poor synthesis, aggregation, loss of activity after immobilization, crosslinking interference, weak printability, cytotoxicity, or poor construct function. This type of structured feedback can support more informative iterative learning. Repeated design-test-learn cycles may gradually identify combinations of sequence features, conjugation strategies, and material conditions associated with robust function. Achieving this goal will require collaboration across computational biology, peptide chemistry, biomaterials, and biofabrication, together with reporting standards that support data reuse and external validation (Clayton et al., 2025; Han, 2025).

### 8.4 Reproducibility, translation, and validation requirements

Reproducibility requires transparent reporting of model, data, material, and validation variables. Reports should specify model version, training data, preprocessing, modality type, fusion strategy, filtering rule, objective weight, uncertainty estimate, material-context variable, and validation level (Collins et al., 2024). Reporting frameworks for AI-based prediction models provide useful principles, although biomaterial peptide design also requires material and fabrication metadata (Park et al., 2024). Data provenance is equally important. Peptide models may combine public databases, private experimental records, synthetic negatives, and material-specific assays (Clayton et al., 2025). For material-aware design, provenance should also cover material sources, fabrication protocols, assay platforms, laboratory conditions, and batch effects, because these variables can substantially influence biomaterial performance. Computational prediction should not be treated as the final endpoint (Perin et al., 2026). Future studies should compare foundation model-assisted workflows with simpler descriptors, conventional machine-learning models, and empirical screening baselines. Prospective validation in predefined material systems provides stronger evidence than retrospective benchmark performance alone. External testing and cross-laboratory replication will be needed to establish practical reliability.

Translation also requires attention to manufacturability, safety, scalability, and regulatory expectations (Li Q. et al., 2025). Peptides intended for biomaterial or biofabrication applications must be reproducibly synthesized, stable during storage and processing, compatible with relevant cells and materials, and safe in the intended biological environment (Velu et al., 2025). Future models should therefore prioritize candidates with realistic translational potential rather than only high predicted activity.

### 8.5 Concluding remarks

Foundation model-assisted peptide screening, generation, and material-aware optimization are moving beyond sequence-based prediction toward integrated design workflows. The forward-looking priority is not simply to make larger or more multimodal models, but to connect molecular predictions with material processing, construct-level validation, and biological function. This requires PLM-based and sequence-level tools to be embedded within material-aware validation, reporting, and feedback systems.

Future progress should ultimately be judged by whether computationally prioritized peptides can be translated into reproducible, bioactive, printable, and regenerative material systems. Achieving this goal will require iterative collaboration across computational modeling, peptide chemistry, biomaterials engineering, biofabrication, and functional tissue engineering. Foundation model-assisted peptide design should therefore be regarded as an enabling component of experimentally grounded material development rather than as a substitute for validation at the material and construct levels.
